# Evidence for Multiple Phototransduction Pathways in a Reef-Building Coral

**DOI:** 10.1371/journal.pone.0050371

**Published:** 2012-12-05

**Authors:** Benjamin Mason, Michael Schmale, Patrick Gibbs, Margaret W. Miller, Qiang Wang, Konstantin Levay, Valery Shestopalov, Vladlen Z. Slepak

**Affiliations:** 1 Department of Molecular and Cellular Pharmacology, University of Miami Miller School of Medicine, Miami, Florida, United States of America; 2 Rosenstiel School of Marine and Atmospheric Science, University of Miami, Miami, Florida, United States of America; 3 National Marine Fisheries Service, Southeast Fisheries Science Center, Miami, Florida, United States of America; 4 Department of Ophthalmology, University of Miami Miller School of Medicine, Miami, Florida, United States of America; University of Delhi, India

## Abstract

Photosensitive behaviors and circadian rhythms are well documented in reef-building corals and their larvae, but the mechanisms responsible for photoreception have not been described in these organisms. Here we report the cloning, immunolocalization, and partial biochemical characterization of three opsins and four G proteins expressed in planulae of the Caribbean elkhorn coral, *Acropora palmata.* All three opsins (acropsins 1–3) possess conserved seven-pass transmembrane structure, and localize to distinct regions of coral planulae. Acropsin 1 was localized in the larval endoderm, while acropsin 2 was localized in solitary cells of the ectoderm. These rod-like cells displayed a remarkably polarized distribution, concentrated in the aboral end. We also cloned four *A. palmata* G protein alpha subunits. Three were homologs of vertebrate Gi, Go, and Gq. The fourth is presumably a novel G protein, which displays only 40% identity with the nearest known G protein, and we termed it Gc for “cnidarian”. We show that Gc and Gq can be activated by acropsins in a light-dependent manner in vitro. This indicates that at least acropsins 1 and 3 can form functional photoreceptors and potentially may play a role in color preference during settlement, vertical positioning and other light-guided behaviors observed in coral larvae.

## Introduction

Nearly every phylum in the animal kingdom contains representatives with at least simple “visual” structures. These are diverse, in both form and function, ranging from the eye-like organelles found in some single-celled dinoflagellates and the ocelli of sponge larvae to the complex camera-type eyes of cephalopods, compound eyes of crustaceans and lens eyes of mammals [1]. Diverse examples of visual structures also exist within phyla and even within single species. One extreme example is found in the box jellyfish *Tripedalia cystophora*. This cnidarian has 24 eyes of four morphological types, including complex camera-type eyes and single-celled, pigmented ocelli, which it possesses during its larval stage [2].

In contrast, all life-stages of reef-building corals (Cnidaria: Anthozoa: Hexacorallia: Scleractinia) lack even basic visual structures, yet notable examples of photosensitive behavior have been described in these organisms [3]. Light influences tentacle expansion and retraction [4], [5], regulates circadian clocks [6], [7], and has been implicated in the synchronization of reproductive timing [8]–[10]. It also influences the behavior of corals’ reproductive propagules, planula larvae. Larvae of some species demonstrate phototaxis [11], [12], vertical migration [13], and settlement preferences based on light quality and/or intensity [12], [14], [15]. The only photosensory molecules described in corals are blue-light sensing cryptochromes [6]. However, we recently reported that coral larvae display red-photosensitivity [16] and preference for red substrates during settlement [15], leading us to hypothesize that long-wavelength sensitivity and color discrimination may be the result of a rhodopsin-based visual system.

The universal photopigment rhodopsin is a protein-chromophore complex consisting of a seven-transmembrane protein, opsin, and a light sensitive chromophore, a derivative of vitamin A, typically 11-cis-retinal. The chromophore is covalently bound to a universally conserved lysine residue located in the seventh transmembrane domain of opsin and resides within a pocket formed by the protein [17]. Interactions between the 11-*cis*-retinal and amino acids lining this pocket act to both stabilize the chromophore and spectrally tune the photopigment [18], [19]. Consequently, the spectral sensitivity of rhodopsins can range from ultraviolet to infrared [20].

**Figure 1:**
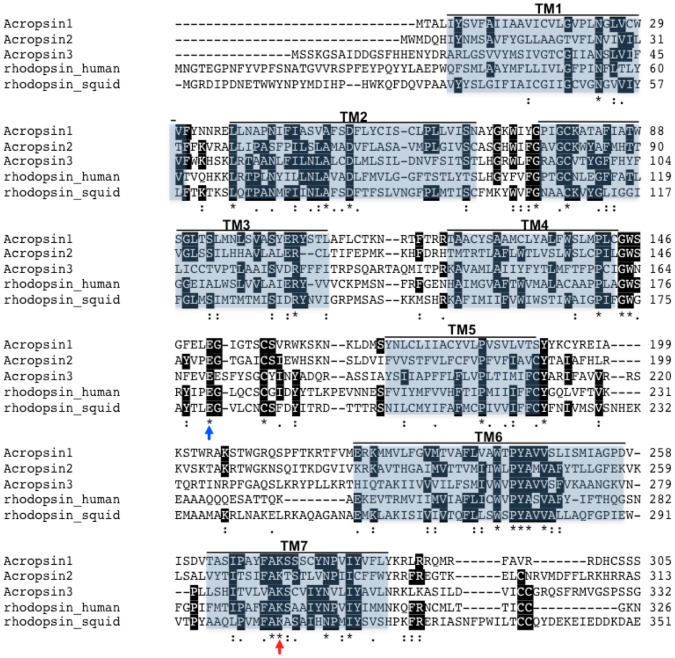
Partial alignment of acropsins 1–3 with human and squid rhodopsins. Amino acid sequences of acropsins 1–3 were aligned with *Homo sapiens* (NP_000530) and *Loligo forbesi* (CAA40108) rhodopsins using ClustalW. Shaded regions indicate transmembrane domains (TM1-TM7) and are based on positions in *H. sapiens* rhodopsin. The red arrow denotes the retinal-binding lysine (K296 in human rhodopsin); blue arrow, the conserved glutamate (E181 in human rhodopsin) that serves as the counterion stabilizing the Schiff base linkage of retinal. Black highlighting indicates identity with the consensus. NOTE: The total length of acropsin 3 is 666 amino acids, but the c-terminus bears no resemblance to other known opsins.

To date, over 1000 opsins have been described from diverse cell types and taxa [21]. For example, in vertebrates, ciliary opsins are expressed in rods and/or cones (specialized neurons in the retina) where they form the molecular basis for vision. Another type of opsin, melanopsin, is also expressed in eyes of vertebrates but not in the rods and cones, and it is not involved in vision. Melanopsin is found in retinal ganglion cells and is responsible for photoentrainment of circadian rhythms and pupillary reflex. In most invertebrates, vision in mediated by rhabdomeric opsins, such as those expressed in the compound eyes of insects and the camera-type eyes of cephalopods. Interestingly, opsins are also found extraocularly (i.e., in organs, glands and tissues of the body) where they are thought to function as irradiance detectors, providing information about the ambient light environment and influencing non-image forming tasks, such as biological rhythms [22]. Several notable examples of extraocular opsins include: pinopsin, found in the pineal gland of some vertebrates; neuropsins, with expression in mammalian neural tissue, eye and brain, but also weak expression in testes and spinal cord; cnidopsins, a family of opsins unique to cnidarians which are expressed in jellyfish eyes but have also been found in neurons and gonadal tissue [21].

Rhodopsins are G protein-coupled receptors (GPCRs), a family of receptors that initiate signaling through heterotrimeric (Gαβγ) G proteins. The interaction of a G protein with an active GPCR results in GDP/GTP exchange by the alpha subunit, followed by dissociation of Gα-GTP from Gβγ complex and subsequent activation of effector enzymes or ion channels. There are four known families of G-protein alpha subunits: Gs, Gi, Gq, and G12 [23]. The alpha subunits Gt and Gq mediate the dominant visual cascades in vertebrates and invertebrates, respectively. Gt, a photoreceptor-specific Gi family member, activates phosphodiesterase, which hydrolyzes cyclic GMP to GMP. This results in the closing of cyclic nucleotide-gated channels and hyperpolarization of the photoreceptor cell. Gq activates phospholipase C, causing the opening of ion channels TRP and TRPL and resulting in photoreceptor depolarization [24]. Recently, other opsin-G protein signaling pathways (Go [25] and Gs [26]) have been discovered in more basal metazoans.

The aim of this study was to determine whether larvae of reef-building corals possess opsin-based phototransduction. Here we report the cloning, cellular localization and initial biochemical characterization of opsins in larvae of the Caribbean elkhorn coral, *Acropora palmata*.

## Materials and Methods

### Sample Collection

*A. palmata* larvae (used for RNA extraction and histology) were raised in the laboratory from field-collected and laboratory-crossed gametes according to previously described methods [27]. Gametes used for fertilization were collected from several reefs (The Elbow, Horseshoe, Sand Island, Molasses Reefs) in Key Largo, FL in August 2006 and again in August 2009. Larvae were 6 days old (post-fertilization) at the time of sampling. A fragment of adult *A. palmata* (approximately 1 cm^2^), collected from Horseshoe Reef (under permit FKNMS-2010-055), provided the material used for immunoblots. Protein lysate was prepared by scraping tissue from the skeleton with a sterile, surgical blade, while collecting the removed tissue in chilled isotonic buffer containing 1× protease inhibitors (Complete, Roche). Protein loading buffer (Laemmli buffer) was added and samples were loaded immediately or aliquotted and frozen at −80°C.

**Figure 2:**
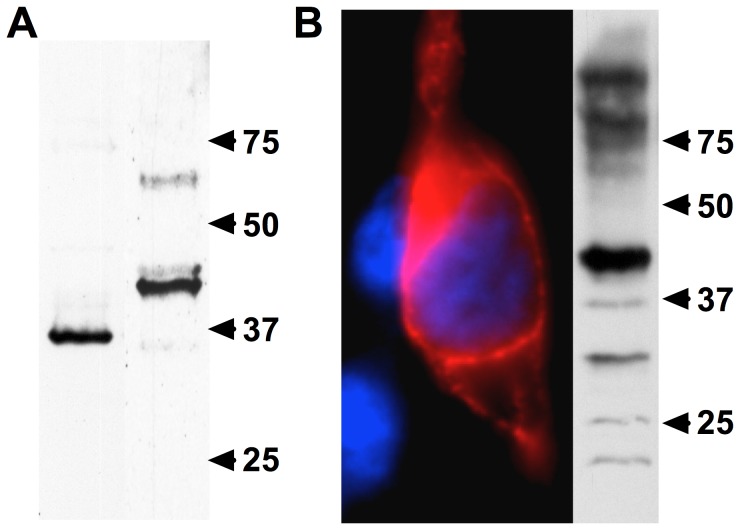
Expression of endogenous and recombinant acropsins. (A) Immunoblot of a total protein lysate obtained from adult *A. palmata* probed with anti-acropsin 1 (∼36 kDa; Lane 1) and acropsin 2 (∼40 kDa; Lane 2) antibodies. (B) Acropsin 2-1D4 chimera was expressed in HEK293t cells as described in Materials and Methods. Left panel: cells were fixed and stained with 1D4 monoclonal antibody (red) and DAPI (blue). Right panel: Western blot probed with 1D4 antibody. The 40 kDa band represents acropsin 2.

### Identification of Opsin- and G Protein-like Transcripts

Known rhodopsin proteins (human (NP_000530) and squid (*Loligo forbesi*; CAA40108)) were used as bait during BLAST (tblastn) searches of *A. millepora* and *A. palmata* larval transcriptomes (http://sequoia.ucmerced.edu/SymBioSys/index.php). Transcripts that encoded open reading frames containing putative opsin domains (seventh transmembrane domains containing a retinal-binding lysine) were considered candidate opsins.

To identify G protein-like transcripts, the *A. millepora* larval transcriptome SymBiosis database was blasted using human G protein alpha subunits (Gi, Go, Gq, Gt) as bait.

**Figure 3:**
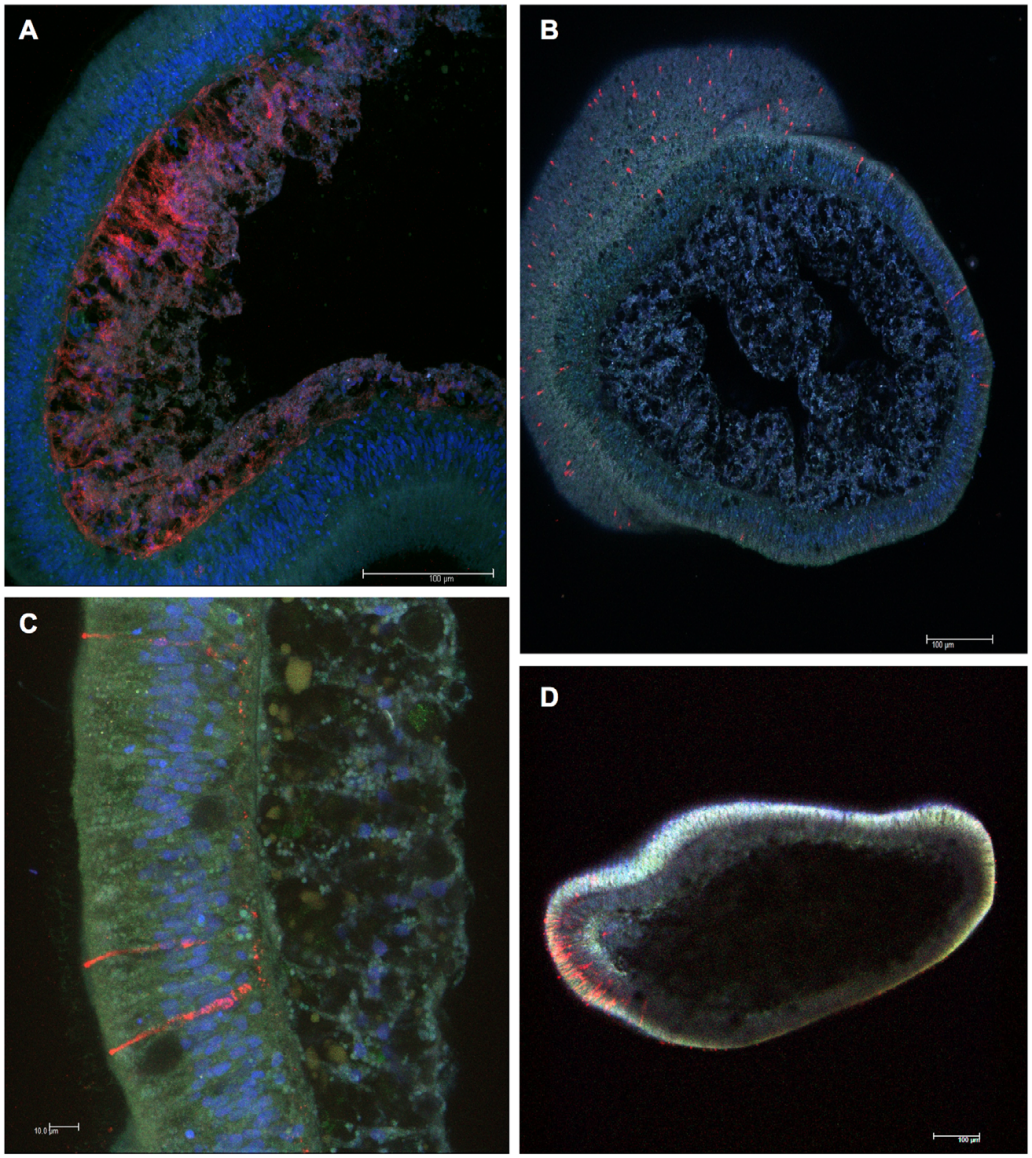
Localization of acropsins 1 and 2 in *A. palmata* larvae. Planulae were fixed, sectioned, probed with anti-acropsin antibodies as described in Materials and Methods. Laser confocal microscopy shows localization of the immunofluorescence (secondary antibody, Cy3, red), endogenous green fluorescence, and DAPI staining of the nuclei (blue). (A) Longitudinal section of the larva probed with anti-acropsin 1 antibody. Positive labeling of acropsin 1 (red) is observed in the larval gastrodermis. Image: snapshot (single z plane); objective = 40×; scale bar: 100 µm. (B) Transverse cross section labeled with anti-acropsin 2 antibody showing localization of acropsin 2 in solitary epithelial cells (red). Image: maximum projection; objective = 20×; scale bar: 100 µm. (C) Morphology of three acropsin 2-positive cells with proximal ends terminating in the mesoglea. Image: maximum projection; objective = 63× oil; scale bar: 10 µm. (D) Longitudinal section of the entire animal showing the predominantly aboral localization of acropsin 2-positive cells. Image: snapshot (single z plane); objective = 10×; scale bar: 100 µm.

### RNA Isolation and cDNA Synthesis

Total *A. palmata* larval RNA was isolated from late-stage (6 day-old) larvae, preserved in RNA*later* tissue storage reagent (Ambion) and frozen at −80°C. Isolation of RNA was achieved by Phenol:Chloroform:IAA, Acid-Phenol:Chloroform extraction following the ToTALLY RNA (Ambion) protocol for samples stored in RNA*later* and frozen at −80°C. Extracted RNAs were precipitated using isopropanol, collected by centrifugation and re-suspended in nuclease-free water.

3′/5′ RACE-ready cDNAs were synthesized by reverse transcription using and M-MLV reverse transcriptase (Clontech). An oligo-dT primer was used for synthesis of 3′ RACE ready cDNAs and either Smarter RACE (Clontech) or RLM RACE (Ambion) kits were used for synthesis of 5′ RACE ready cDNAs.

**Figure 4:**
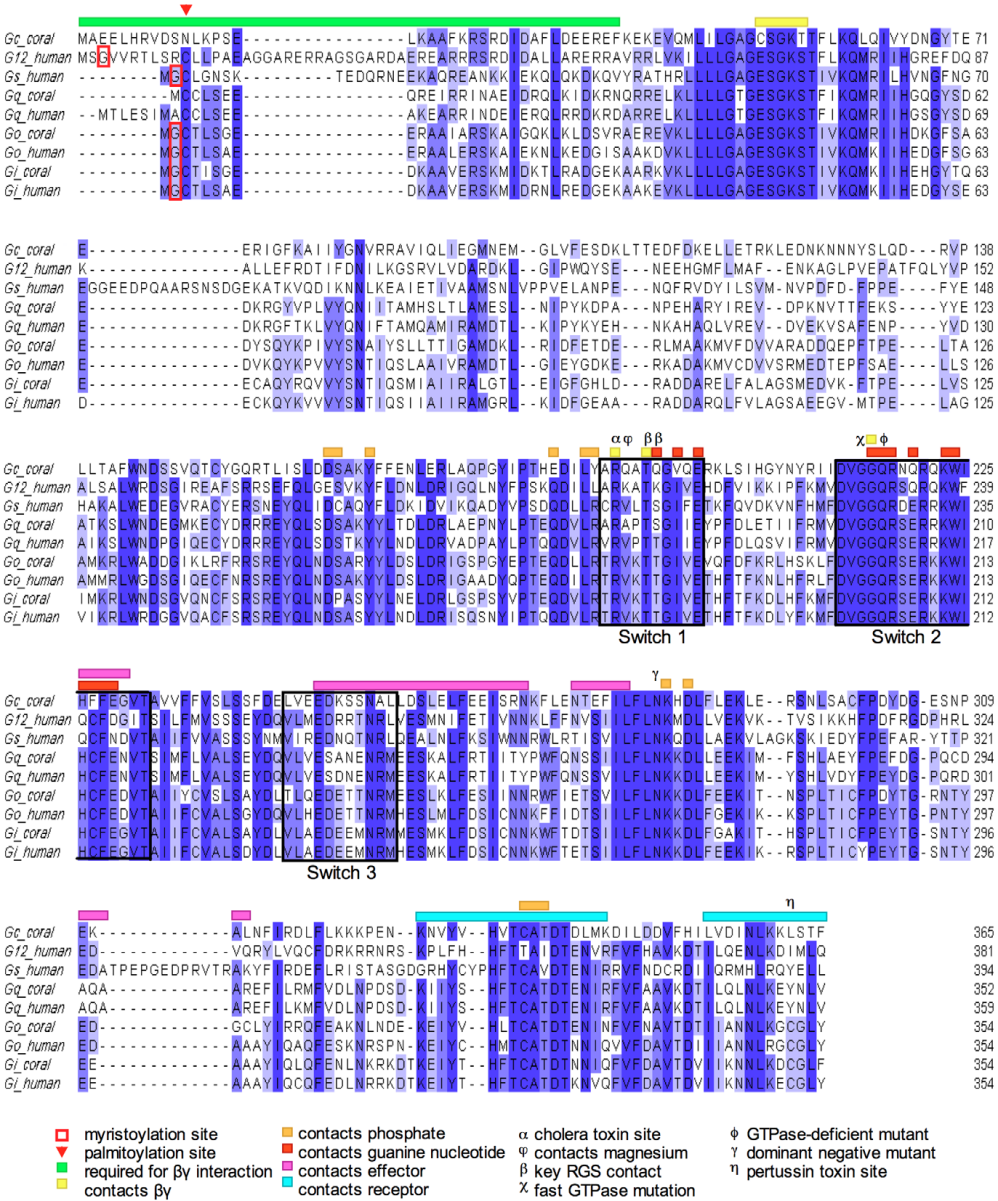
Annotated alignment of coral and human G protein alpha subunits. Alignment of four coral G protein alpha subunits (Gc_coral (JQ966103),Gi_coral (JQ966104), Go_coral (JQ966105), and Gq_coral (JQ966106)) with representatives of the human G alpha subunit families - human Gi, Go, Gq, Gs, and G12. (Gi_human (P04899.3), Go_human (P09471.4), GNAQ_human (P50148.4), GNA12_human (Q03113.4)). The alignment was constructed using MUSCLE (48) and decorated in Jalview (49). Shading indicates identity. Switch regions are indicated by black boxes. Other functional residues and domains (50,51) are indicated, as described in the key.

### 3′/5′ RACE

Nested, gene-specific RACE primers were designed (using Primer3; [28]) from candidate opsin transcripts identified in the *A. millepora* or *A. palmata* transcriptomes and used to amplify the corresponding gene products from *A. palmata* larval 3′ RACE-ready cDNA. PCR products were gel-purified and sequenced directly (Genewiz) or cloned first and then sequenced. Consensus sequences were determined and edited using DNAStar, Lasergene V7, SeqBuilder application.

### Cloning

RACE products that were not sequenced directly were cloned into TOPO pCR2.1 cloning vectors by overnight incubation at room temperature (RT) with T4 DNA ligase. The resulting ligation products were used to transform *E. coli* (electrocompetent DH5α; Invitrogen) and grown overnight on LB (Kan30) agar plates. Inserts were sequenced using M13R and M13F(−47) universal primers (Genewiz).

Full-length acropsin cDNAs were cloned into pcDNA3.0 (Invitrogen) or pMT4 mammalian expression vectors. The acropsin cDNAs were also tagged by addition of the bovine rhodopsin 1D4 epitope (TETSQVAPA) to their C-termini. Reverse PCR primers containing the nucleotide sequence encoding this epitope and forward primers (above) were used to amplify and subclone the 1D4 constructs. In the case of acropsin 3, the 1D4 construct was truncated by removal of the c-terminus, so that the length of the resulting c-tail was equivalent in length to bovine rhodopsin. Truncation of the c-terminal tail has been shown to enable their expression of some opsins (invertebrate and melanopsins with unusually long tails), that otherwise are not expressed in mammalian cells [29].

**Figure 5:**
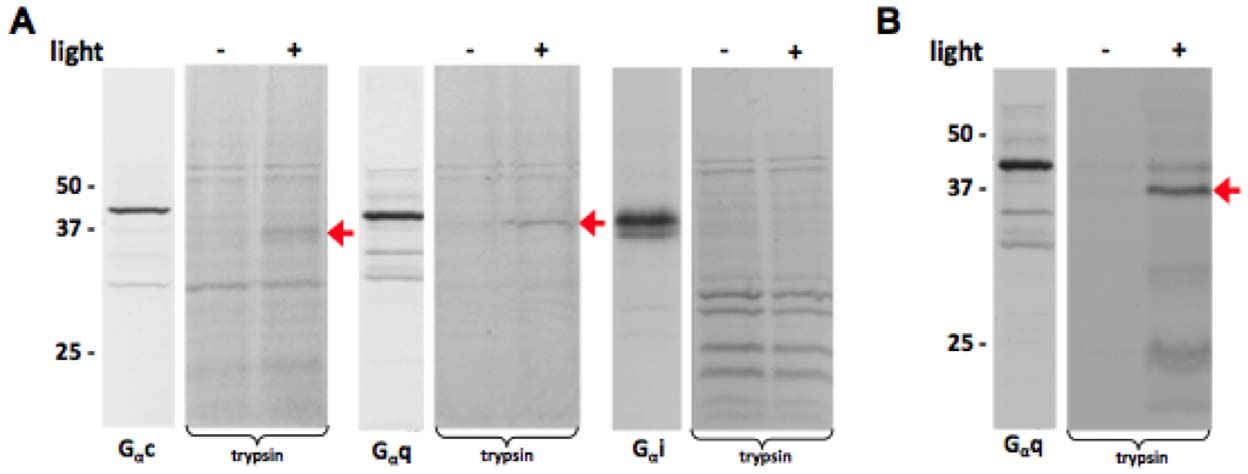
Light-dependent activation of coral G proteins by acropsins. In vitro translated Gα subunits were reconstituted with membranes of acropsin-expressing HEK293t cells as described in Materials and Methods. The proteins were exposed to light or kept dark and then incubated with trypsin for 60 min. The resulting patterns of ^35^Met-labeled products were analyzed by SDS-PAGE. (A) Reconstitution of acropsin 1 with coral Gc, Gq, and Gi; GTPγS was present in all reactions. Red arrows indicate stabilized forms of Gc (∼36 kDa) and Gq (∼37 kDa) in light. (B) Reconstitution of acropsin 3 with coral Gq. The 37 kDa product, observed only in the presence of light, is indicated by the red arrow.

### Antibody Production and Purification

Rabbit polyclonal antisera were raised against synthetic peptides designed from regions of Acropsin 1 and 2. Two peptides (corresponding to the third cytoplasmic loop and c-terminal tail regions) were selected for each Acropsin. Peptides were synthesized by Genscript and antibody production performed by Cocalico Biologicals. Peptides were conjugated to keyhole limpet hemocyanin (KLH) and mixed with Complete Freund’s Adjuvant for initial inoculation and Incomplete Freund’s Adjuvant for subsequent boosts. Antibodies were affinity purified using an Aminolink Plus Immobilization Kit (Piercenet) and dialyzed overnight at 4°C against 3000× volume of phosphate-buffered saline (PBS). Antisera against the acropsin 1 peptide DNYSTRNRPENI, corresponding to the c-terminal 12 amino acids, and against the acropsin 2 third cytoplasmic loop peptide NSQITKDGVIVKRK showed greatest reactivity and specificity and were used in the study.

### Immunofluorescence Confocal Microscopy

Larvae were fixed in 4% paraformaldehyde. Samples were rinsed with PBS and embedded in 3.5% agar in 1× PBS. 150 micron sections were cut using a Lancer® Vibratome, Series 1000 Sectioning System. Sections were permeabilized in PBS containing 0.15% TWEEN20 and 0.15% TRITON for 20 minutes, incubated in blocking solution (5% goat serum; 2% BSA; 0.1% TWEEN; 1× PBS) for 45 minutes, washed in PBS +0.15% TWEEN and stained with rabbit-polyclonal antibodies diluted 1∶300 in solution for antibody (2% BSA; 0.15% TWEEN20; 1× PBS). Sections were incubated overnight at 4°C, washed 3×30 minutes in PBS containing 0.15% TWEEN. After washing, sections were incubated for 2 hours with Cy-3 (Jackson Immuno Research) goat anti-rabbit IgG diluted 1∶1000. Sections were washed as above and incubated with DAPI diluted 1∶10,000 in PBS containing 0.15% TWEEN20 or mounted onto microscope slides using Fluoromount with DAPI (Southern Biotech). Preparations were examined using a Leica Inverted and Upright TCS SP5 Confocal Microscopy System, with a 4 fluorescent channel detection system. Images were captured and processed using the Leica software (Leica Application Suite).

### Expression and Reconstitution of Acropsins with 11-cis-retinal

Acropsin-1D4 fusion proteins were transiently expressed in HEK293t cells. Cells (10^7 ^per 10 cm diameter petri dish) were transfected using TransIT-LT1 Transfection Reagent (Mirus Bio LLC). Cells were grown at 37°C with 5.0% CO_2_ for 24 h, collected in buffer A (HEPES, pH 6.8) with 1× protease inhibitors (Complete, Roche) on ice and transferred to Litesafe (Argos) microcentrifuge tubes. Cells were vortexed and incubated overnight at 4°C with an excess of 11-cis-retinal. Prior to assays cells were lysed by repeated cycles of freeze-thaw at −80°C and passage through a 23G needle. Membranes were then collected by centrifugation at 14 K rpm for 30 min at 4°C. All preparations were done in the dark or under dim red light.

### Limited Trypsinolysis of G Protein Alpha Subunits

G protein alpha subunits were cloned into the pcDNA3.0 expression vector (Invitrogen; as above), and the TNT T7 Quick-Coupled Transcription/Translation System (Promega) was used to produce ^35^S-methionine labeled proteins. Two microliters of the TNT reactions, containing *in vitro* translated ^35^S-methionine-labelled G protein alpha subunits, were added to aliquots of prepared HEK293t membranes (representing approximately 10^6^ cells) containing acropsins. The reactions were incubated with 100 µM GDP in the dark for 30 min after which GTPγS was added to a final concentration of 10 µM. Reactions were illuminated for 5 min using a halogen lamp with a fiber optic light guide. Tubes were then centrifuged and the supernatant was transferred to a clean tube, mixed with phenylalanyl chloromethyl ketone (TPCK)-treated trypsin (Sigma), resolved by SDS PAGE, and transferred to nitrocellulose paper. Digested coral G protein alpha subunits were detected by autoradiography, following overnight exposure of film at −80°C.

## Results

We cloned full-length cDNAs encoding three opsins – acropsins 1–3 (Genbank: JQ966100-JQ966102) from *A. palmata* planulae. Two of these are within the size range of known opsins (34–50 kDa), but the third is uniquely large. *Acrop1* contains a 1.073 kb open reading frame (ORF) that encodes a 343aa protein with a predicted molecular weight of 38.7 kDa, and the *Acrop2* ORF (1.053 kb) encodes a 349aa protein with a predicted molecular weight of 39.5 kDa. In contrast, *Acrop3,* contains a 1.998 kb ORF that encodes a 666aa (74.3 kDa) opsin (Fig. 1).

Acropsins 1–3 are most similar to putative opsins found in the sea anemone *Nematostella vectensis* genome (*Nemop* groups 1–3 within the Cnidopsins; [30]; Fig. S1) but also possess features that are highly conserved among both vertebrate and invertebrate opsins. All three acropsins contain the universally conserved lysine residues within their predicted seventh transmembrane domains, and glutamic acid residues in their second extracellular loop regions (Fig. 1). The lysine is found in the identical position in all known opsins and serves as the site of attachment of 11-cis retinal [17], [31], while the conserved glutamic acid serves as a counterion, balancing the positive charge created by the protonated Schiff base [32], [33].

To study expression of acropsin 1 and 2 in coral planulae, we raised rabbit polyclonal antibodies against synthetic peptides. These antibodies detected native and overexpressed proteins (Figs. 2 and 3). On immunoblots, the protein bands corresponded to the predicted molecular weights of acropsin 1 (∼38 kDa) and acropsin 2 (∼40 kDa) (Fig. 2A). Immunofluorescence microscopy of fixed larvae showed that anti-acropsin antibodies labeled distinct cell populations. Acropsin 1 was confined to the larval endoderm (Fig. 3A), while acropsin 2 was localized in a sub-population of solitary epithelial cells. Cells expressing acropsin 2 were scattered throughout the larval ectoderm but were much more abundant in the aboral end of the planulae (Fig. 3B–D). The acropsin 2-positive cells contain axon-like processes that terminate in the larval “nerve region” [34] (Fig. 3C; Fig. S2). This morphology resembles that of other photosensory cells and also suggests that these cells are neurons. For example, cells found in the ocellus of another cnidarian, the hydroid *Leuckartiara octona*, consist of solitary, monociliated ectodermal neurons, with a corona of microvilli surrounding each cilium and their basal ends forming axons [35], [36]. Mono-ciliated columnar epithelial cells containing coronae of microvilli are common in the ectoderm of coral planulae; in other cnidarians, similar cells posses neurosecretory activity and are hypothesized to have sensory functions [37].

We also cloned *A. palmata* cDNAs encoding four G protein alpha subunits (Genbank accession numbers JQ966103–JQ966106). According to reciprocal best BLAST hits against the *Acropora millepora* larval transcriptome, three of these are orthologs of human Gq, Gi, and Go, displaying 71–84% identity to human counterparts. The forth G alpha subunit lacks a human ortholog and displays only 39–40% identity with the known invertebrate and vertebrate G protein alpha subunits. Based on blastp of the UniprotKB/Swiss-Prot database, it contains domains responsible for GTP and Mg^2+^ binding, switch regions, and sites responsible for interaction with Gßy and the receptor (Fig. 4), but may interact with a novel effector. Interestingly, this Gα lacks the key Gly and Cys residues within the N-terminus that are responsible for N-myristoylation and palmitoylation (Fig. 4), thus it is not obvious how this protein binds to the membranes. We named this G protein Gc for “cnidaria”.

For the initial analysis of acropsin function, we expressed them in HEK293t cells, isolated membranes, and reconstituted these with 11-cis retinal in darkness. Coral G proteins were expressed in vitro and labeled with ^35^S-Met, using a rabbit reticulocyte system. These lysates were combined with the membranes containing acropsins. To detect G protein activation, we used limited trypsinolysis, an assay based on the increased proteolytic stability of G protein α subunits bound to GTPγS [38], [39]. Our tests showed that in the presence of GTPγS and light some coral G proteins formed characteristic ∼37 kD products. Acropsin 1 facilitated stabilization of Gc and coral Gq (Fig. 5A). Acropsin 1/Gc signaling may represent a novel phototransduction pathway. Illumination of acropsin 3 resulted in the formation of a stable product with only one of the coral G proteins, Gq (Fig. 5B). In the absence of light, stable trypsinolysis products were not present. These results lead us to conclude that acropsin 1 and acropsin 3 can activate coral G proteins in a light-dependent manner.

## Discussion

This study provides initial evidence for the existence of multiple phototransduction pathways in corals. Acropsin 3 interacted specifically with a coral ortholog of mammalian Gq. Existence of a Gq-coupled phototransduction pathway in Cnidaria provides new insight into the evolution of phototransduction. Two contrasting hypotheses currently exist. One argues that phototransduction evolved from a non-opsin GPCR using a cyclic-nucleotide-gated (CNG) pathway and that Gq phototransduction (the dominant visual pathway among protostomes, and non-visual pathway among deuterostomes; [21]) evolved after the Cnidarian-bilaterian split [40]. The other, based on the sequence diversity and phylogenetic position of sea anemone (anthozoan) opsins, suggests an earlier divergence of the Gt and Gq pathways, even prior to the Cnidarian-bilaterian split [30]. Our results are consistent with the latter hypothesis. In addition, phospholipase C (PLC) and protein kinase C (PKC) genes, involved in the Gq/inositol phospholipid signaling, are found in sponges (poriferans) and *Hydra* (a hydrozoan) [41], indicating the origin of Gq signaling predates the parazoan-eumetazoan split. Furthermore, BLAST searches of coral (larval) transcriptome databases retrieve sequences encoding a TRPC channel (the type of ion channel involved in Gq phototransduction). For example, a transcript (Transcript ID: aug_v2a.09061.t1; http://marinegenomics.oist.jp) from *Acropora digitifera* larvae is highly similar (E = 1.0e−86) to melanopsin-specific, TrpC3 channel from *Rattus norvegicus*. Thus, it appears likely that either: (1) a Gq phototransduction cascade evolved prior to the Cnidarian split and was subsequently lost in Hydrozoans, or (2) Gq phototransduction has evolved twice - once in Anthozoans and a second time prior to the protostome/deuterostome split.

We did not observe G protein activation by acropsin 2. It is possible that this opsin interacts with coral Gs or G12, which are found in the *Acropora millepora* larval transcriptome (e.g., Gs: contig10430 and G12: contig3459; http://sequoia. ucmerced.edu/SymBioSys/index.php). While these G proteins are not involved in traditional phototransduction pathways (i.e., Gt-vertebrate/ciliary and Gq-invertebrate/rhabdomeric; [24]), the box jellyfish *Carybdea rastonii* was recently shown to possess a Gs-coupled opsin [26]. Another explanation could be the instability of acropsin 2 pigment under our experimental conditions or another limitation of our assay system. Finally, acropsin 2 may not activate G proteins but instead function as the RGR/peropsin family of opsins (Fig. S1), which are known to behave as photoisomerases, converting all-trans retinal to its 11-cis form [42], [43].

The localization of acropsins may offer insight into their potential function in the larva. Do they measure irradiance, reporting on the time of day or lunar cycle phase, or do they play a role in light-guided behavior? Acropsin 1-positive cells, localized in the larval endoderm, lack obvious structural organization. This suggests that acropsin 1 cannot determine the directionality of light and thus is unlikely to function in larval phototaxis. It is noteworthy that cryptochromes - blue-light photosensors involved in circadian clocks - are also localized in these tissues [6]. Perhaps acropsin 1 behaves in a manner similar to vertebrate melanopsin [44] and plays a role in photoentrainment. In contrast, the strikingly polarized expression of acropsin 2 (Fig. 3D) causes us to speculate that this receptor plays a role in phototaxis. The aboral epidermis is the sensory end of coral planulae, and the heterogeneous cell types of this region display neurosecretory activity [34]. Planulae generally swim with the aboral epidermis forward and use it to explore the substrate as they seek appropriate settlement habitat [45]. Phototaxis requires directional light sensing and, in marine invertebrate larvae, typically involves organization of photoreceptors into at least simple eyespots [46]. The concentration of acropsin 2-positive cells in the aboral ectoderm indicates the presence of specific photoreceptor-like cells and may confer directional light sensing. Additional biochemical characterization and determination of acropsin absorbance spectra would offer further insight into the functions of the acropsins in coral planulae.

## Supporting Information

Figure S1 **Opsin family tree.** Phylogenetic tree consisting of fifty-six amino acid sequences representing eight opsin families. The phylogeny was constructed with CLUSTAL 2.1 using a neighbour-joining method with distance correction (after [47]). The cnidarian opsins (cnidopsins) form distinct clades and within these, the anthozoan opsins (acropsins 1–3 and *Nematostella vectensis*) occur in three groups, distinct from each other and the jellyfish (cubozoan) opsins. These are similar to Groups 1–3, previously described for *N. vectensis* opsins (27). Acropsins 1 and 2 cluster with Nvop Group 1 while acropsin 3 is most similar to members of Nvop Group 3. The following Genbank accession numbers correspond to the sequences used in the construction of Fig. 2 (Opsin family tree): Nvop37.1 (FAA00413.1), Nvop40.1 (FAA00384.1), Nvop209.3 (FAA00399.1), Acrop2 (JQ966101), Nvop202 (FAA00389.1), Nvop273 (FAA00392.1), Acrop1 (JQ966100), PcopB (BAF95843.1), PcopC (BAF95844.1), CropK2 (BAF95841.1), CropJ (BAF95842.1), CropG1 (BAF95845.1), CropN2 (BAF95846.1), Scallop_OPSD2 (O15974.1), *Branchiostoma*_Amphiop1 (BAC76019.1), *Branchiostoma*_Amphiop2 (BAC76020.1), Nvop29 (FAA00401.1), Acrop3 (JQ966102), Nvop241(FAA00396.1), Human_neuropsin Neuropsin1 (NP_859528.1), Mouse_neuropsin (DAA01972.1), Squid_retinochrome (CAA40422.1), Chicken_RGR (AAR02099.1), Mouse_RGR (AAC69836.1), Human_RGR (AAA56748.1), Human_peropsin (DAA00976.1), Chicken_peropsin (NP_001073227.1), Zebrafish_peropsin (NP_001004654.1), *Branchiostoma*_Amphiop3 (BAC76023.1), Spider_peropsin (BAJ22674.1), *Branchiostoma*_Amphiop6 (BAC76024.1), Zebrafish_melanopsin (AAL82577.1), Mouse_melanopsin (AAF24979.1), Human_melanopsin (NP_001025186.1), Cow_melanopsin (NP_001179328.1), Squid_rhodopsin (CAA40108.1), Tubeworm_r-opsin (CAC86665.1), *Drosophila*_OPS6 (O01668.1), Crayfish_opsin (AAB25036.1), Lobster_opsin (ABI48884.1), *Drosophila*_NinaE (AAA28733.1), *Drosophila*_opsin (AAA28735.1), *Drosophila*_Rh5 (AAB38966.1), *Daphnia*_sw-opsin (EFX75461.1), *Daphnia*_uv-opsin (EFX81332.1), Zebrafish_rhodopsin (NP_571159.1), Human_rhodopsin (NP_000530.1), Bovine_rhodopsin (NP_001014890.1), *Xenopus*_rhodopsin (NP_001080517.1), Chicken_rhodopsin (NP_001025777.1), Sea_urchin_encephaopsin (BAH28806.1), Zebrafish_TMT (NP_001112371.1), Pufferfish_TMT (AAL83430.1), Zebrafish_pan/enceph (NP_001104634.1), Human_panopsin (AAK37447.1), Mouse_pan/enceph (NP_034228.1).
(TIF)

Figure S2 **Localization of acropsin 2 in epithelial cells of** ***Acropora palmata*** **larvae.** Immunofluorescent confocal image of a section showing morphology of acropsin 2-positive epithelial cells. Cell nuclei stained by DAPI are shown in blue, green represents endogenous GFP fluorescence, and red shows labeling by anti-acropsin 2 primary and Cy3-labeled secondary antibody. Objective = 63× oil; scale bar = 10 µm.
(TIF)
