# Supplementary figures and images for: Evidence for Multiple Phototransduction Pathways in a Reef-Building Coral
Source: PLoS One. 2012 Dec 5;7(12):e50371. doi: 10.1371/journal.pone.0050371 (PMC3515558; doi:10.1371/journal.pone.0050371)

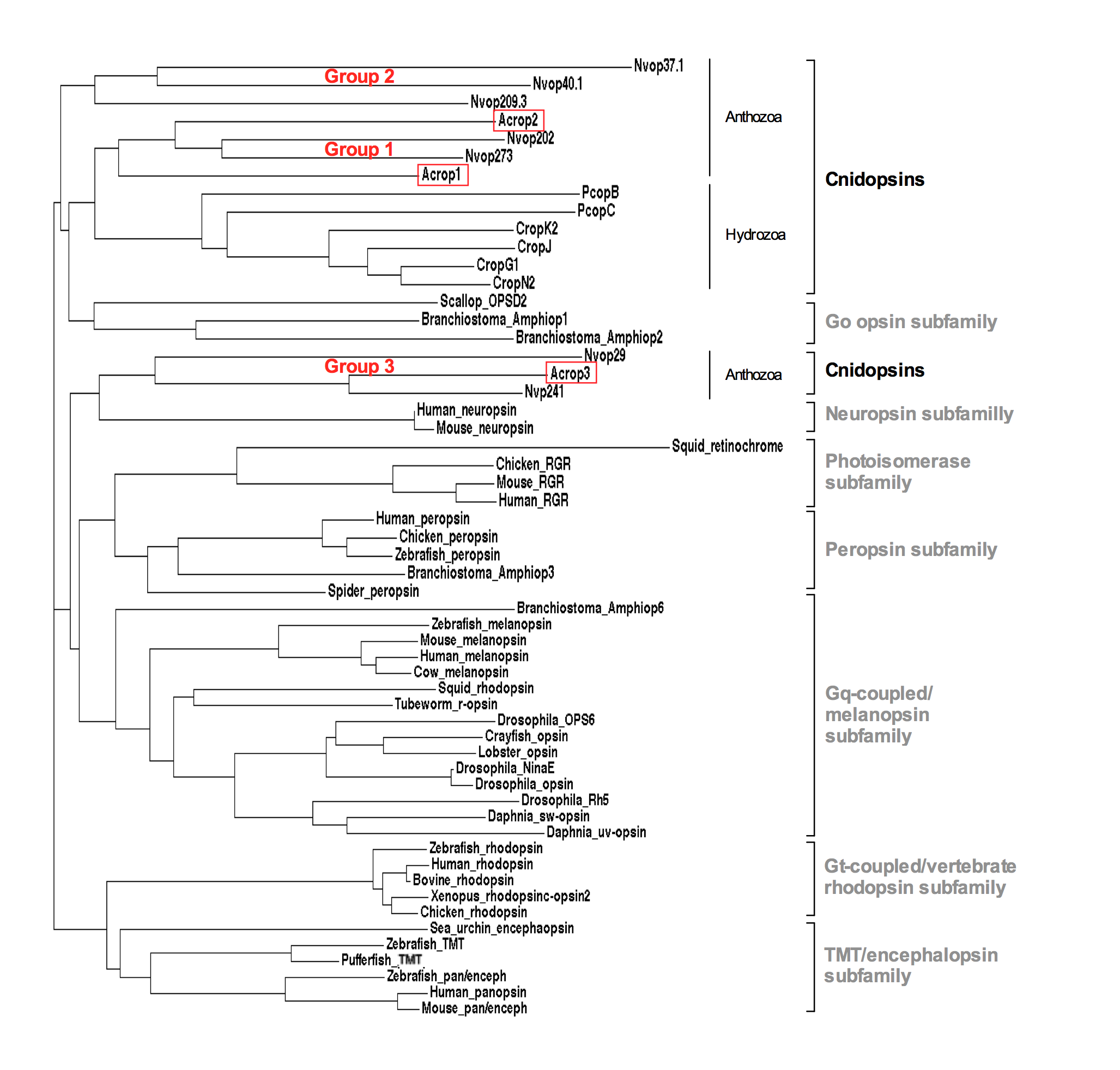

Supplement: Figure S1 — Opsin family tree. Phylogenetic tree consisting of fifty-six amino acid sequences representing eight opsin families. The phylogeny was constructed with CLUSTAL 2.1 using a neighbour-joining method with distance correction (after [47]). The cnidarian opsins (cnidopsins) form distinct clades and within these, the anthozoan opsins (acropsins 1–3 and Nematostella vectensis) occur in three groups, distinct from each other and the jellyfish (cubozoan) opsins. These are similar to Groups 1–3, previously described for N. vectensis opsins (27). Acropsins 1 and 2 cluster with Nvop Group 1 while acropsin 3 is most similar to members of Nvop Group 3. The following Genbank accession numbers correspond to the sequences used in the construction of Fig. 2 (Opsin family tree): Nvop37.1 (FAA00413.1), Nvop40.1 (FAA00384.1), Nvop209.3 (FAA00399.1), Acrop2 (JQ966101), Nvop202 (FAA00389.1), Nvop273 (FAA00392.1), Acrop1 (JQ966100), PcopB (BAF95843.1), PcopC (BAF95844.1), CropK2 (BAF95841.1), CropJ (BAF95842.1), CropG1 (BAF95845.1), CropN2 (BAF95846.1), Scallop_OPSD2 (O15974.1), Branchiostoma_Amphiop1 (BAC76019.1), Branchiostoma_Amphiop2 (BAC76020.1), Nvop29 (FAA00401.1), Acrop3 (JQ966102), Nvop241(FAA00396.1), Human_neuropsin Neuropsin1 (NP_859528.1), Mouse_neuropsin (DAA01972.1), Squid_retinochrome (CAA40422.1), Chicken_RGR (AAR02099.1), Mouse_RGR (AAC69836.1), Human_RGR (AAA56748.1), Human_peropsin (DAA00976.1), Chicken_peropsin (NP_001073227.1), Zebrafish_peropsin (NP_001004654.1), Branchiostoma_Amphiop3 (BAC76023.1), Spider_peropsin (BAJ22674.1), Branchiostoma_Amphiop6 (BAC76024.1), Zebrafish_melanopsin (AAL82577.1), Mouse_melanopsin (AAF24979.1), Human_melanopsin (NP_001025186.1), Cow_melanopsin (NP_001179328.1), Squid_rhodopsin (CAA40108.1), Tubeworm_r-opsin (CAC86665.1), Drosophila_OPS6 (O01668.1), Crayfish_opsin (AAB25036.1), Lobster_opsin (ABI48884.1), Drosophila_NinaE (AAA28733.1), Drosophila_opsin (AAA28735.1), Drosophila_Rh5 (AAB38966.1), Daphnia_sw-opsin (EFX75461.1), Daphnia [file pone.0050371.s001.tif]

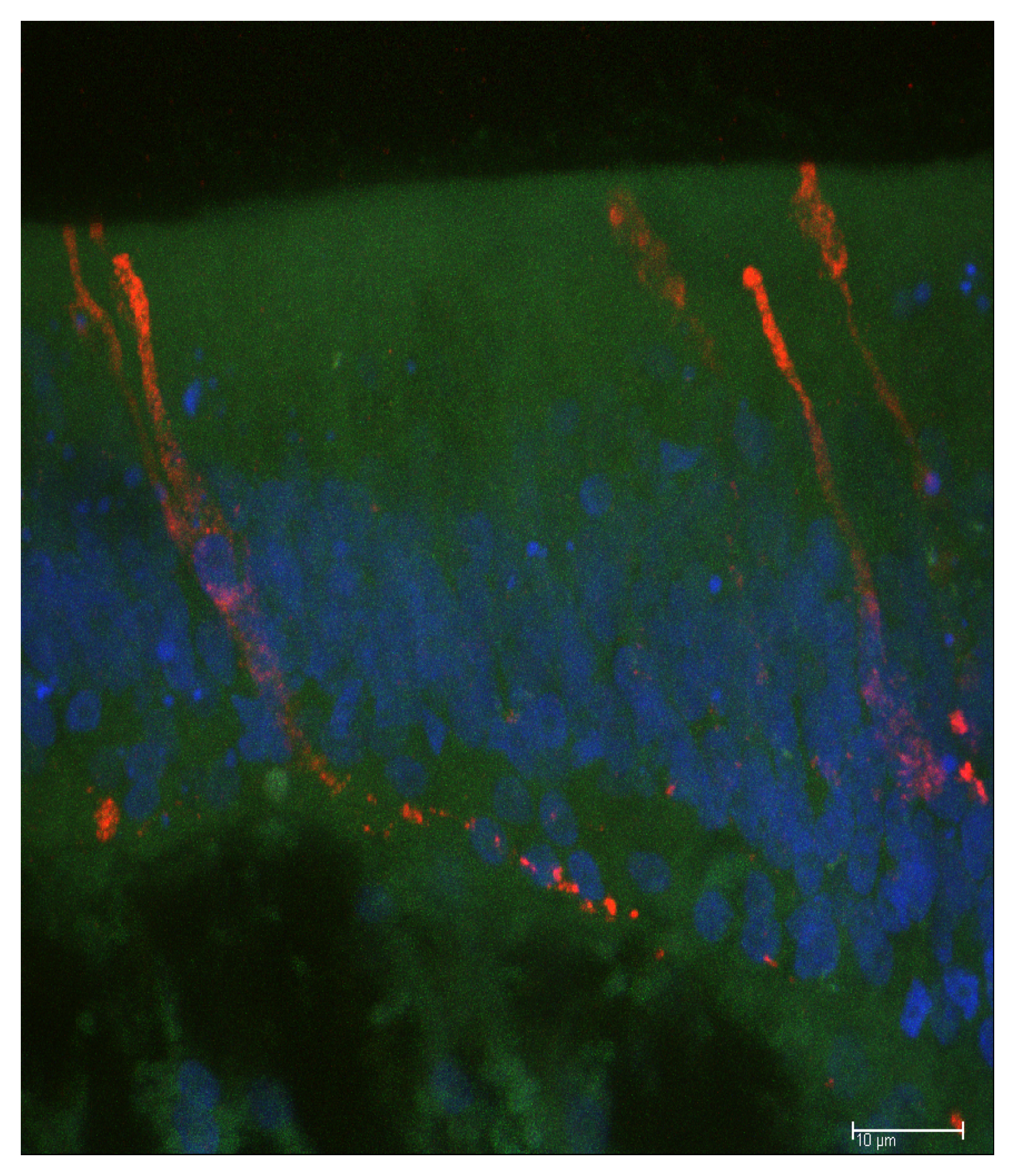

Supplement: Figure S2 — Localization of acropsin 2 in epithelial cells of Acropora palmata larvae. Immunofluorescent confocal image of a section showing morphology of acropsin 2-positive epithelial cells. Cell nuclei stained by DAPI are shown in blue, green represents endogenous GFP fluorescence, and red shows labeling by anti-acropsin 2 primary and Cy3-labeled secondary antibody. Objective = 63× oil; scale bar = 10 µm. (TIF) [file pone.0050371.s002.tif]
